# Preoperative Templating for Total Hip Arthroplasty: A Method for Calibrating Digital Radiographs Using Patient Demographics and Anthropometric Measurements

**DOI:** 10.7759/cureus.47668

**Published:** 2023-10-25

**Authors:** Joseph Attwood, Philippa Banks, Adam Sidhom, Hemant Pandit, Sameh Sidhom, Bernard van Duren

**Affiliations:** 1 Trauma and Orthopaedics, Huddersfield Royal Infirmary, Huddersfield, GBR; 2 Orthopaedics, Leeds Institute of Rheumatic and Musculoskeletal Medicine, University of Leeds, Leeds, GBR

**Keywords:** anthropometric measurements, digital radiograph, preoperative templating, neck of femur fractures, sub capital hip fracture, total hip athroplasty, total joint arthroplasty

## Abstract

Background

Preoperative templating aids the surgeon in estimating implant size and placement. Calibration markers are used to set the correct magnification of digital images before templating. Improper marker placement or complete absence can lead to inaccuracy or an inability to calibrate images altogether.

Aims

This study describes a method for calibrating images using a patient’s femoral head size (FHS) predicted using demographics and anthropometric data.

Materials and methods

A formula predicting the FHS was derived from a cohort of 507 patients who underwent hemiarthroplasty for an intracapsular fractured neck of the femur through multivariate regression analysis. A separate validation cohort (n=50) who had undergone total hip arthroplasty (THA) had postoperative radiographs calibrated using the predicted FHS and the native contralateral hip as a surrogate calibration marker. The THA femoral head implant size was subsequently measured and compared with the actual implant size selected intraoperatively. Measurements were performed by two independent assessors to determine intra- and interobserver reliability.

Results

Multivariate regression analyses showed four variables significantly correlated with the size of the femoral head: gender (p < 0.001), height (p < 0.001), weight (p < 0.001), and race (Asian) (p = 0.01). Using these, a regression model to predict the FHS was obtained with an R^2^ value of 0.65 and a standard error of 2.18 mm. The validation cohort showed that THA head implant size could be accurately measured with an average root-mean-squared error (RMSE) of 1.41 mm (SD = 0.97 mm; %RMSE = 4.7%). The implant head size was measured to be within 5%, 10%, and 15% RMSE in 57.5%, 93.0%, and 100.0% of cases, respectively. There was excellent intraobserver (R^2^ = 0.94 and 0.95) and interobserver (R^2^ = 0.94) reliability.

Conclusions

The novel method proposed and validated in this study, using a predicted FHS to calibrate digital images, provides an alternative means of templating THA for fractured neck of the femur patients, in whom external calibration markers are often absent.

## Introduction

Preoperative templating is the process by which the surgeon predicts the size of implants required and determines their optimal placement based on preoperative imaging. The reliable and accurate use of preoperative templating for total hip arthroplasty (THA) is widely adopted by surgeons [1-3]. It assists the surgeon in replicating hip biomechanics and reducing complications, including leg length discrepancy, implant wear, dislocation, and periprosthetic fracture [4,5].

Traditionally, templating was done using acetate overlays on radiographs, with fixed magnification [6]. In recent decades, the widespread use of Picture Archiving and Communication Systems (PACS) has facilitated a transition to digital templating techniques that make use of an external calibration object to calculate a magnification factor. A radiopaque sphere or 'marker ball' of a known size is the most commonly used [7], although other methods are available (e.g., Kingmark [8]). Unfortunately, templating for arthroplasty in fractured neck of the femur (NOF) patients is hindered by challenges in correctly placing the calibration object.

The accuracy of digital templating systems using a fixed calibration object depends on the proper placement of the marker at the level of the trochanter and fully within the field of view. Incorrect placement of the calibration marker (CM) in both medial-lateral and anteroposterior planes results in magnification errors [9,10]. Variations in magnification due to incorrect placement of the external CMs and larger-sized individuals have also been reported [7,9]. In the context of fractured NOF patients, placement is complicated by patients being in pain with their injury, thereby preventing correct marker placement. Failure to request a CM when acquiring preoperative radiographs often results in markers being omitted altogether [11,12].

Previous studies have explored ways to overcome the problem of inadequate calibration. The use of a fixed magnification factor is an alternative option when no CM is available. Studies have shown that it can be used accurately; however, it is a device- and institution-specific method [8,12,13]. Polishchuk et al. proposed a formula based on gender, height, age, and race to predict the native femoral head size (FHS) [14]. Although a solution based on patient demographic data that is not reliant on radiographs offers an attractive alternative, perhaps for predicting hemiarthroplasty implant size, it has limitations when applied to THA. Digital image templating provides additional information regarding femoral offset and the relationship of the implant to anatomical landmarks to inform component placement (e.g., the size of the implant relative to the femoral canal to ensure fit and the distance from the greater trochanter to implant shoulder, which can be useful in restoring leg length), as well as planning an appropriate cement mantle.

A robust method to obtain accurate magnification for templating in the absence of a CM would be a useful tool for surgeons who require an alternative to poorly placed or absent CMs when performing THA in fractured NOF patients. The aim of this paper was to develop and test a calibration method based on patient demographic data that can be used in the absence of a CM or when one is poorly placed.

## Materials and methods

The study was registered with and approved by the clinical governance department of our hospital as a service evaluation project. TraumaCad (BrainLab, Munich, Germany) allows digital image calibration by one of three options: (1) Automatic, in which a CM ball of known size is automatically detected and the image is calibrated accordingly. In our institution, a standardized 25.4 mm diameter ball is used. (2) Manual calibration, in which, typically, a CM is measured by the user with a ruler or circle tool and provided the size is known the image can be calibrated. (3) Oversize, in which the image scale can be increased or decreased by a specified percentage based on the user’s preference. In cases where a CM is not available, our institution uses a fixed magnification of 125%.

We proposed a novel method for calibrating digital pelvic radiographs using the native femoral head as a surrogate CM. Based on the findings of a previous study [14], patient demographics and anthropometric measurements can accurately predict the FHS. Our method involves manually templating the femoral head circumference using the circle tool in the absence of a CM. Image magnification is then calculated using the predicted FHS, as calculated below, rather than the standardized 25.4 mm diameter of the calibration ball marker. This method produces an image calibrated based on demographic and anthropometric data.

To investigate the accuracy of our proposed methodology, our study was undertaken in two stages: (1) develop a model to predict FHS and (2) test using the predicted FHS in combination with native femoral head as a CM.

Stage 1: predicting FHS

To obtain an equation for predicting FHS based on patient demographics and anthropometric measurements relevant to our patient population, we employed the method described by Polishchuk et al. [14]. A retrospective dataset was collected to serve as a training dataset. The data were collected for patients who underwent hemiarthroplasty between January 1, 2018, and September 22, 2020. For each patient, data on height, weight, gender, race, side, age, and size of the femoral head or implant used in surgery were recorded. Using this training dataset, a multivariate regression analysis (ANOVA) was conducted using all recorded variables to establish which were significant. After identifying the significant variables, these were incorporated into a regression model to predict FHS. Regression analyses were performed using Microsoft Excel (Microsoft Corporation, Redmond, Washington).

Stage 2: calibration accuracy evaluation

To assess the accuracy of using the native femoral head as a CM, we evaluated a unique cohort of 50 patients who had undergone THA for a fractured NOF at our institution between August 1, 2019, and June 6, 2021. Gender, height, weight, and race were recorded to allow the predicted FHS to be calculated using the predictive formula determined in Stage 1. The images were then calibrated using the native femoral head (contralateral side) as the CM, employing the manual calibration tool of the TraumaCAD software. Once the image had been calibrated, the caliper tool was used to measure the diameter of the femoral head implant. The measured size of the femoral head was then compared with the actual implanted FHS obtained from the operative records. Measurements were performed independently by two observers (PB and JA) to assess both intra- and interobserver reliability. This was done for all 50 consecutive patients on two separate occasions one week apart. Observers were blinded to their previously recorded measurements.

Statistical Analysis

The sizes measured using native femoral head calibration were compared to the implant sizes used during the operation. To analyze agreement visually, a Bland-Altman analysis was used to illustrate the limits of agreement [15]. If agreement is good, then the differences should be randomly scattered around the zero-difference reference line. To assess the accuracy of the proposed calibration method, the calculated implant head sizes were compared to the actual head sizes recorded intraoperatively using Lin's Concordance Correlation Coefficient (CCC) [16]. Lin's CCC tests the concordance between a new test or measurement (new calibration measurement) and a gold standard test or measurement (intraoperative/actual implant head size). Both inter- and intraobserver correlations were calculated to quantify the possible effect of our measurement methods on the results presented. The calculations were performed using Microsoft Excel and R (R Core Team, Vienna, Austria).

## Results

Stage 1: predicting FHS

Training Data 

The training data consisted of 507 patients who underwent hemiarthroplasty for a fractured NOF between January 1, 2018, and September 22, 2020. The cohort included 359 women and 148 men, with 274 left and 233 right hemiarthroplasties performed. The mean height was 163 cm (SD: 10, range: 124-190), and the mean weight was 61 kg (SD: 14, range: 31-110). The majority of the cohort (452) were White-British, and the remainder were distributed among Asian (8), White-Other (18), Other-Not Stated (29), and Black (1).

Multivariate Regression Model 

Multivariate regression analyses showed that four variables significantly correlated with the size of the femoral head. These included gender (p < 0.001), height (p < 0.001), weight (p < 0.001), and race (Asian) (p = 0.01). Based on the determined significant variables the following regression model to predict FHS was obtained with an R^2^ value of 0.65 and a standard error of 2.18 mm.

FHS = 25.84 + 4.07(G) - 2.11(A) + 0.11(H) + 0.03(W)

where G is the gender (0 for woman and 1 for man), H is the height in centimeters, W is the weight in kilograms, and A stands for Asian (1 for Asian and 0 for all others).

**Figure 1:**
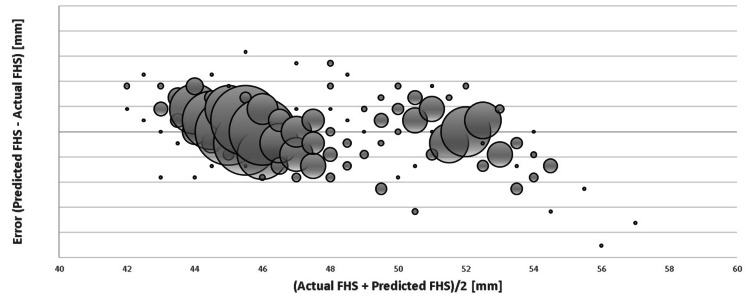
Bland-Altman plot showing the margin of error (predicted – actual FHS), where 1 SD = 2.21 mm on the y-axis plotted against the average FHS ((actual + predicted)/2). Bubble sizes are representative of the number of values.

A comparison of the predicted FHS using the above formula to the actual FHS measured at operation for the training data (n=507) is shown in the Bland-Altman style plot in Figure 1.

**Figure 2:**
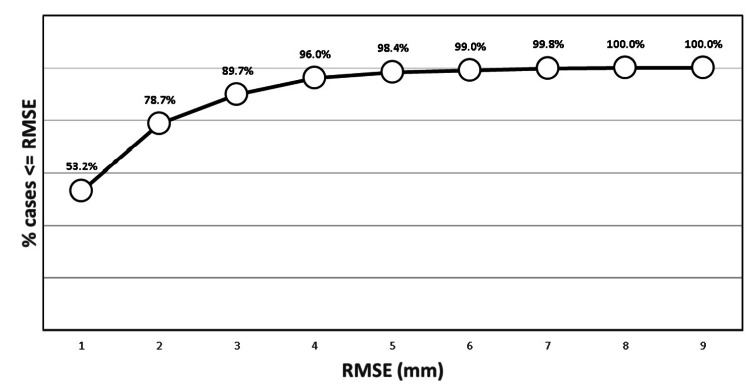
A plot showing the percentage of cases (n/507) within (≤) RMSE limits, i.e., 53% of predicted values were within or equal to 1 mm.

The formula predicted the FHS to be 1, 2, 3, 4, 5, 6, 7, 8, 9, and 10 mm in 53.2, 78.7, 89.7, 96.0, 98.4, 99.0, 99.8, 99.9, and 100.0% of cases, respectively (Figure 2).

Stage 2: calibration accuracy evaluation

Fifty consecutive patients who underwent a primary THA for a fractured NOF, presenting to our institution between August 1, 2019, and June 9, 2021, were identified. The group consisted of 37 women and 13 men; 22 had a right-sided THA. The mean height was 166 cm (SD: 9, range: 148-182), and the mean weight was 69 kg (SD: 18, range: 37-140).

**Figure 3:**
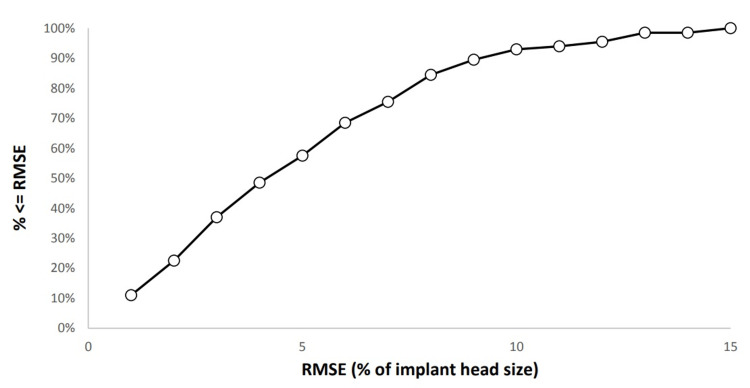
A plot showing the percentage of measurements (n/200) within (≤) %RMSE limits, i.e., 57.5%, 93.0%, and 100% of the predicted values were within or equal to 5, 10, and 15 RMSE. RMSE, root-mean-squared error.

For all measurements undertaken by both observers (n = 200), comparison to the actual FHS (femoral head measurements using the contralateral femoral head as a CM were compared to the THA femoral head used intraoperatively) resulted in an average root mean squared error (RMSE) of 1.41 mm (SD: 0.97 mm) (Table 1). This equated to an average RMSE of 4.7% (SD: 3.3%, 95% CI <5.6%). Using the predicted FHS to calibrate the image meant that the implant head size was measured to within 5, 10, and 15% RMSE in 57.5, 93.0, and 100.0% of cases, respectively (Figure 3).

**Table 1:** Overview of the data showing the comparison of measured FHS using the contralateral femoral head and predicted FHS calibration with the actual implant head size. This is shown as separate cohorts for 28 mm and 32 mm heads as well as RMSE and %RMSE for all measurement comparisons. FHS, femoral head size; RMSE, root-mean-squared error.

	28 mm	32 mm	RMSE	%RMSE
Mean	29.67	32.31	1.41	4.72
SD	0.98	1.37	0.97	3.32
Range	27.9-32.15	29.38-35.13	0.01-4.15	0.05-14.81
Upper 95% CI	30.10	31.81	1.70	5.69
Lower 95% CI	29.24	32.80	1.13	3.75

**Figure 4:**
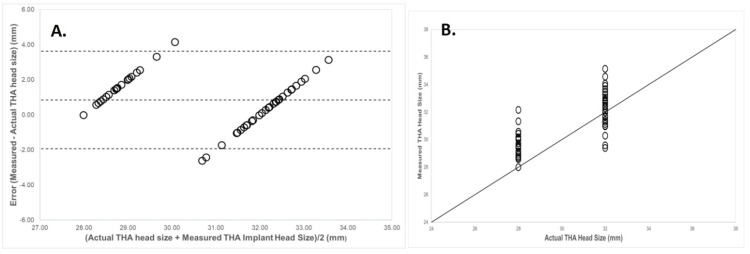
(A) Bland-Altman plot displaying the difference between total hip arthroplasty femoral head implant size measured using our proposed method and the actual implant size used at operation. (B) Plot showing the relationship between measured THA implant size and actual implant size. The line represents perfect correlation. THA, total hip arthroplasty.

The implant head sizes used intraoperatively were either 28 mm (n=20) or 32 mm (n=30). Measurements of implant FHS on FHS-calibrated images showed mean averages of 29.7 mm (95% CI 29.3-30.2) for the 28 mm cohort and 32.2 mm (95% CI 31.7-32.8) for the 32 mm THA head sizes. The Bland-Altman plot measurement of implants on calibrated images showed values congregated around the 28 mm and 32 mm points, as these were the only possible femoral head implant sizes (Figure 4). The mean difference between measured and actual sizes was 0.85 mm, with 1.96 SD limits of agreement between -1.9 mm and 3.6 mm. Lin's CCC comparing the head size measured using the contralateral head as a CM to the actual head size (gold standard) showed a correlation of 0.64 (95% CI 0.56-0.71).

The measurements made by the observers were highly reproducible upon repeated attempts. The intraobserver Pearson's correlation coefficients were 0.94 (95% CI 0.90-0.97) and 0.95 (95% CI 0.91-0.97) (Table 2). The interobserver reliability was 0.94 (95% CI 0.91-0.94).

**Table 2:** Intra- and Interobserver reliability of the native femoral head calibration method. Data from two independent observers who demonstrated this method to be highly reproducible. RMSE, root-mean-squared error.

	Intraobserver Agreement		Interobserver Agreement
	Observer 1 (PB)	Observer 2 (JA)	Observer 1 vs Observer 2
Mean RMSE	1.34	1.48	-
SD RMSE	0.91	0.95	-
Upper 95% CI	1.61	1.76	-
Lower 95% CI	1.08	1.21	-
Pearson's correlation	0.94	0.95	0.94
Upper 95% CI	0.90	0.91	0.91
Lower 95% CI	0.97	0.97	0.97
n	50	50	50

## Discussion

This paper presents a novel method for calibrating digital pelvic radiographs using demographic and anthropometric data. We have used regression analysis to show that FHS could be predicted to within 5 mm of that measured intraoperatively in 98% of cases, which represents a 10.5% error based on mean FHS. Subsequently, the use of the native femoral head as a substitute CM measured the implant FHS with an RMSE of 1.34 mm (4.7%). Measurement was within a 10% error in 93% of cases, and all measurements were within a 15% error. This demonstrates that using a combination of the predicted FHS and the native femoral head as a CM is a viable calibration technique in the absence of a CM, or when a CM is poorly placed, as often occurs in fractured NOF patients.

The use of external CMs has been shown to be accurate in studies examining THA [7,8,17-19]. Unfortunately, inaccuracies can arise from poor placement of the marker [2,20]. In the context of hip fractures, CMs are often suboptimally positioned, or radiographs are taken with no CMs present [11,12]. This is due to several factors: unknown diagnosis at the time the radiograph is taken, patients experiencing too much pain, patients being confused and agitated, and occasionally, inexperienced radiology staff. Parwaiz et al. [12] highlighted this by reporting that only 20% of radiographs for possible fractured NOF included a CM at audit, and only managed to achieve 76% following three cycles of stakeholder education, poster placement, and an electronic prompt on the X-ray machine. However, osteoarthritis remains the main indication for 91.5% of THAs that occur in the UK [21]. The elective setting allows the opportunity for repeat radiographs if CMs are omitted due to human error. However, this creates additional demands on resources, further exposure to radiation, and inconvenience for the patient. The images are also subject to the same potential magnification errors that occur from inadequate positioning in the coronal and sagittal planes [22].

The use of a fixed magnification factor also allows for digital templating in the absence of a CM and has been widely used in THA [2,23,24]. Franken et al. [2] and Archibeck et al. [23] compared fixed magnifications of 121% with the CM method; both concluded that fixed magnification was equally good, if not superior. Franken et al. [2] used a similar method of using postoperative THA radiographs and measured implant head size. They found an RMSE of 1.42% with fixed magnification, compared with 2.55% with CM calibration, both better than the 4.7% error that we reported for our method. Brew et al. [24] used a fixed magnification of 119.8% with an RMSE of 0.6 mm (0.5%) on the measurement of FHS on postoperative THA radiographs. They also reported excellent intra- and interobserver reliabilities, both at 0.993. Although fixed magnification appears to be extremely accurate, it should be noted that magnification may vary substantially between radiology departments and devices [12,13]. This means that published magnification values may not be applicable, and departments wanting to use a fixed magnification would have to calculate site-specific values. Additionally, with the use of modern adjustable equipment, it may be difficult to ensure consistent patient placement within the radiographic field. Another disadvantage of using a fixed magnification becomes more apparent in high- or low-BMI patients, where the plane of the hip may differ substantially from the mean. FHS calibration uses height and weight in its calculation, and therefore, this would be accounted for in our model. Further studies directly comparing FHS calibration to already established methods such as a CM and fixed magnification would be useful to determine the most accurate and reliable technique.

The regression model was obtained with an R^2^ value of 0.65, similar to that published by Polishchuck et al. [14], who reported an R^2^ value of 0.68 for FHS based on a smaller cohort of 100 patients who had undergone hemiarthroplasty. They reported gender, age, race, and height as significant variables in their model, whereas in this study, we found gender, race, height, and weight to be significant. Interestingly, age was not found to be significant in our cohort, whereas weight was significant in this study but not in their cohort. Other studies have looked at predicting implant sizes in total knee replacement based on demographic and anthropometric measurement data and have variously found gender, height, weight, age, and race to be significant [25-28]. There may remain other demographic or anthropometric variables not yet identified that could improve the accuracy of the predictive formula.

This work has some limitations. The height and weight measurements may be of variable accuracy due to different individuals taking these measurements and the lack of formal calibration of scales. It should be acknowledged that obtaining height and weight measurements for patients with a fractured NOF is difficult due to their pain and inability to stand. As such, the values documented for height and weight in the electronic record used in this study are likely to have been obtained from previous admissions, gleaned from general practitioner records, reported by the patient, or estimated. This represents an "as treated" scenario in which the surgeon would use available or documented information. The regression model used is based on the demographics presenting to our institution. The formula used highlighted a significant correlation between Asian ethnicity and head size; it is conceivable that there may be additional disparities in head size related to race (e.g., a Chinese population may have a different head size compared to an Afro-Caribbean population), which we were unable to detect in our regression analysis due to low numbers. This study focused solely on the accuracy of using the FHS calibration method to measure THA femoral head implant size. We did not template a cohort of patients de novo using our templating method, and prospective studies of this nature would be useful for further validation of this technique. Furthermore, as this study sought only to evaluate the accuracy of using the native femoral head as a CM, other aspects of templating were not considered in this study, including acetabular component size, femoral stem size, and offset.

Future work involving larger and more comprehensive patient cohorts would provide a more accurate prediction of FHS and, subsequently, more precise calibration. Further validation studies would offer valuable information for exploring the more widespread use of the proposed method and its application to preoperative templating for THA.

## Conclusions

In conclusion, this study presents a method that uses a predicted FHS to calibrate pelvic radiographs by employing the native femoral head as a surrogate CM. This novel method was accurate to within 10% RMSE in 93% of cases and 15% RMSE in all cases. Overall, the accuracy of the method presented here was inferior to that reported in the literature for fixed magnification and CM methods. However, if these two options are unavailable, it offers a reasonable alternative. This method does not require the calculation of a site- or machine-specific magnification factor, is less susceptible to out-of-plane errors in patients with high or low BMI, and is independent of the use of an external calibration device. This method provides an alternative calibration technique that can overcome some limitations encountered with the current practice of using a CM, such as inadequate positioning or complete absence, issues that often arise when templating for fractured NOF patients.
